# Aligned silver nanowire-based transparent electrodes for engineering polarisation-selective optoelectronics

**DOI:** 10.1038/srep19485

**Published:** 2016-01-18

**Authors:** Byoungchoo Park, In-Gon Bae, Yoon Ho Huh

**Affiliations:** 1 Department of Electrophysics, Kwangwoon Univ., Wolgye-Dong, Nowon-gu, Seoul 139-701, Korea

## Abstract

We herein report on a remarkably simple, fast, and economic way of fabricating homogeneous and well oriented silver nanowires (AgNWs) that exhibit strong in-plane electrical and optical anisotropies. Using a small quantity of AgNW suspension, the horizontal-dip (H-dip) coating method was applied, in which highly oriented AgNWs were deposited unidirectionally along the direction of coating over centimetre-scale lengths very rapidly. In applying the H-dip-coating method, we adjusted the shear strain rate of the capillary flow in the Landau-Levich meniscus of the AgNW suspension, which induced a high degree of uniaxial orientational ordering (0.37–0.43) of the AgNWs, comparable with the ordering seen in archetypal nematic liquid crystal (LC) materials. These AgNWs could be used to fabricate not only transparent electrodes, but also LC-alignment electrodes for LC devices and/or polarising electrodes for organic photovoltaic devices, having the potential to revolutionise the architectures of a number of polarisation-selective opto-electronic devices for use in printed/organic electronics.

Nanowires are a type of low-dimensional nanomaterial, which like nanoparticles, nanotubes, and nanorods, have attracted great interest because of their excellent physical and chemical properties thanks to their high surface-to-volume ratio, well defined one-dimensional geometry, and high crystallinity, as well as their potential applications^1^,^2^,^3^,^4^,^5^,^6^, in electronics, photonics, and sensors^7^,^8^,^9^,^10^,^11^. Given the current need for transparent, conducting, and flexible layers, metallic nanowires^12^,^13^,^14^, carbon nanotubes^15^,^16^, and/or graphene^17^,^18^ have all seen active use in material science, information, energy, and even in medical fields^19^,^20^. In particular, silver nanowire (AgNW) films allow excellent transmittance and conductance and have therefore been proposed as a candidate material for a transparent and flexible electrode to replace the brittle indium tin oxide (ITO) traditionally used in optoelectronic devices such as touch screen panels, organic light-emitting diodes (OLEDs), and photovoltaic (PV) cells^21^. To promote further improvement of their electronic and optical properties, AgNWs must not only have a high aspect ratio, but must also be preferentially oriented^2^, yielding novel anisotropic properties. For instance, oriented AgNWs are excellent substrates for exciting surface plasmons (SPs), additionally making them good candidates for polarised surface-enhanced Raman spectroscopy^22^,^23^ and/or photonic or guide fibre applications^24^,^25^. However, given the inherent difficulties of producing a uniform alignment of AgNWs, the geometrical advantages of a one-dimensional morphology have not been fully realised, necessitating additional processing steps to achieve the desired ordering of the AgNWs^26^,^27^. To overcome such challenges, several techniques have been developed, including Langmuir-Blodgett^28^, fluid flow^23^,^29^, suspension evaporation^30^, blown bubble^31^, electrospinning^32^, mechanical force^33^, and dip-coating^34^. Nevertheless, despite some successes in all these methods, it remains to be seen whether assembly can be scaled up to large substrates for use in various practical appications. The uniform alignment of nanowires over a large area is therefore a considerable challenge, as is their simple and rapid processing^6^,^34^,^35^.

In this report, we show how the capillary flow seen in a Landau-Levich meniscus of an AgNW suspension during a simple horizontal dip (H-dip) coating process^36^,^37^,^38^,^39^,^40^ generated sufficient strain to align the AgNWs with a high degree of uniaxial orientational ordering on a solid surface. This method is readily expandable to large-scale AgNW networks with processing durations of a few seconds, making them particularly suitable for use in polarisation-selective printed/organic electronic devices.

## Experimental

To fabricate AgNW films on solid substrates we used AgNWs suspended in isopropyl alcohol (IPA) (0.5–2.0 wt%) (NTC-01, Nanopyxis Co., LTD.), with an average diameter and length of about 25 nm and 32 μm, respectively. The AgNWs were synthesised using a modified polyol process^41^. After 400 mL of propylene glycol (PG) as both solvent and reducing agent was heated to 126 °C, a capping agent of polyvinyl pyrrolidone (12 g) as a dispersant was added and dissolved with vigorous stirring until clear, and then halide compounds of KBr (0.2 g) and AgCl (1.0 g) were simultaneously injected into the hot PG media as catalysts. After 90 min, AgNO_3_ (4.6 g) as a metal precursor was dissolved in PG (100 ml) to obtain an AgNO_3_ solution, which was then added to the reaction mixture. After about 30 min AgNWs started to form, and the reaction then continued for about 1 hour to allow complete formation of the AgNWs. The reaction mixture was then cooled to room temperature and the AgNWs were purified by precipitate extraction and resuspension in ethanol, the product then being dispersed and suspended in IPA at an appropriate concentration at room temperature. The AgNW suspension can be considered as quasi-stable over 3 hours, and within this time the dispersion and mass fraction of the AgNWs showed no obvious changes.

**Figure 1:**
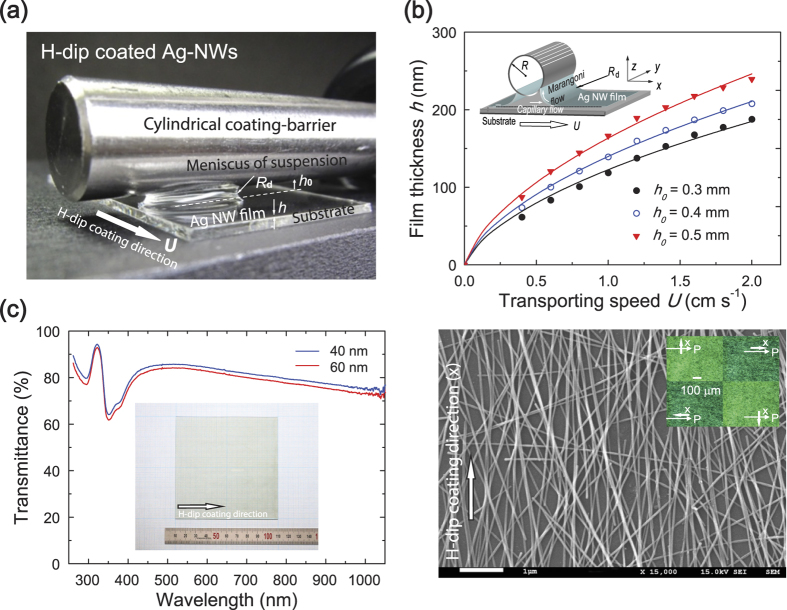
(**a**) Photograph of a Landau-Levich meniscus of an AgNW suspension durin*g the H-dip-coating process* with a gap height *h*_0_ and coating speed *U*. (**b**) Film thickness data of the H-dip-coated AgNW films as a function of coating speed *U* for three different values of *h*_0_. The solid curves show theoretical predictions. The inset shows a schematic diagram of a Landau-Levich meniscus of an AgNW suspension. (**c**) Left: UV-Vis transmission spectra of the H-dip-coated 40 and 60 nm-thick-AgNW films. The inset shows a photograph of an H-dip-coated AgNW film (60 nm) on a 10 × 10 cm^2^ substrate. Right: SEM image of the AgNW film (60 nm) on glass substrate. The inset shows microscopic textures of the H-dip-coated 60 nm-thick-AgNW film at four angles of rotation of the film under a polariser. The white arrows indicate the coating (*x*) direction and the polarising axis of the polariser (P).

As shown in Fig. 1a, the AgNW suspension was coated on glass substrates using the H-dip-coating method^36^,^37^,^38^,^39^,^40^. A standard cleaning procedure was adopted in which the glass substrates were first cleaned ultrasonically in baths of detergent and alcohol for 30 min, then rinsed several times using deionised water, and were then dried using nitrogen followed by ultra-violet (UV) ozone treatment. On the glass substrate, a thin AgNW film was solution-coated using an H-dip coater; the apparatus used for this had a maximum work space of 10 cm × 10 cm. A small volume of AgNW suspension (~5 μl) per unit of coating area (1 cm × 1 cm) was fed into the gap of the cylindrical H-dip-coating head using a syringe pump (NE-1000, New Era Pump Systems Inc.). The height of the gap *h*_*0*_ was adjusted vertically using micrometer positioners mounted at the end of the coating head, and the carrying speed *U* was controlled by a computer-controlled translation stage (SGSP26-200, Sigma Koki Co., Ltd). After a concave meniscus of coating suspension had formed on the substrate, the substrate was transported horizontally such that the meniscus formed by the coating head caused the suspension spread evenly on the transporting substrate while maintaining the shape of the meniscus of the suspension. The AgNWs coated on the glass substrates were then annealed at 120 °C in a dry air environment for 5 s. For comparison, a reference layer of AgNWs was also prepared using spin-coating on the bare glass substrates and then baked at 120 °C for 5 s to extract the residual solvent.

The microscopic morphology of the AgNWs was observed by field emission scanning electron microscopy (SEM, Model JSM-6700F, JEOL Co.). The surface roughness and topographic properties of the AgNW surfaces were characterised using atomic force microscopy (AFM, Nanosurf easyscan2 Nanosurf AG Switzerland Inc.). The macroscopic arrangement of AgNW arrays was observed using optical microscopy (Model BA300Pol., Motic Co.). The optical properties of the AgNWs were investigated using a UV-visible spectroscopy system (8453, Agilent). The sheet resistance of the AgNW film as prepared was also tested using a four-point probe method with a sheet resistivity meter (SRM-232–2000, range 0 to 2000 Ω/square, Guardian Manufacturing).

To fabricate the LC cells, we used a nematic LC mixture (ZLI-2293, Merck), which had a nematic phase below the clearing point of 85 °C. At room temperature the extraordinary (*n*_e_) and ordinary (*n*_o_) refractive indices of ZLI-2293 are 1.6313 and 1.4990 (λ = 589.3 nm), respectively. An empty LC cell was fabricated by sandwiching two glass substrates together; in this case their inner surfaces were coated only with the transparent conductive AgNW films (*ca*. 40–60 nm thick), i.e., without any further ITO electrode or alignment layer. As a result the LC molecules interacted directly with the AgNWs. The cell gap was maintained using glass spacers 4.9 μm thick. The H-dip-coating directions (*x*-directions) of the two AgNW layers on the substrates were set to be parallel or perpendicular to each other. By capillary-filling the empty cells with ZLI-2293 in its isotropic phase (~90 °C), we obtained nematic LC cells after cooling to room temperature using a micro-furnace (FP90 and 82, Mettler Toledo). The LC cell was placed between a polariser at the input end and an analyser at the output end, forming a LC device; illuminating light was normally incident on the LC device. For normally white (NW) mode operation, the polarisation axis of the polariser was set to be parallel to the LC director of the twisted nematic LC (TN-LC) cell at the input end, and the passing axis of the analyser was set perpendicular to the passing axis of the polariser. For normally black (NB) mode operation, the passing axis of the analyser was set parallel to the passing axis of the polariser. The optical characteristics of each component of the LC cells were investigated using polarised microscopy with the UV-visible spectroscopy system.

Organic PV (OPV) cells on glass substrates were fabricated using an inverted structure configuration^37^. An ITO layer (80 nm, 30 Ω/square) on a glass substrate used as the transparent cathode was ultrasonically cleaned using a sequence of detergent, deionised water, acetone, and IPA. The ITO cathode was modified by a ZnO electron-collective layer (ECL, thickness: ~70 nm), prepared by the sol-gel process using a ZnO precursor. The details of the preparation of the ZnO precursor are similar to those described in earlier experiments^37^. Next, a thin cesium carbonate (Cs_2_CO_3_) electron-selective buffer layer (thickness: *ca*. 10 nm) was also spin-coated on top of the ZnO ECL using a solution of Cs_2_CO_3_ (0.2 wt%)^37^. A blended solution of poly[[9-(1-octylnonyl)-9H-carbazole-2,7-diyl]-2,5-thiophenediyl-2,1,3-benzothiadiazole-4,7-diyl-2,5-thiophenediyl] (PCDTBT, 0.456 wt%, 1-material Chemscitech, Inc.) and [6,^6^]-phenyl C_71_ butyric acid methyl ester (PCBM_70_, 1.824 wt%, Nanostructured Carbon, Inc.)^42^ in a solvent of monochlorobenzene was then spin-coated on the ZnO/Cs_2_CO_3_ layers. The PCDTBT:PCBM_70_ bulk-heterojunction (BHJ) PV layer was about 85 nm thick. After spin coating, the PV layer was annealed at a temperature of 65 °C for 1 hour. In order to form a hole-collection layer (HCL) on the PV layer, a *ca*. 20-nm-thick molybdenum oxide (MoO_3_, Aldrich) layer was prepared using thermal deposition at a rate of 0.05 nm/s under a base pressure below 2.7 × 10^−4 ^Pa. Finally, to fabricate a transparent AgNW anode, the prepared AgNW suspension was deposited onto MoO_3_ HCL using the H-dip-coating method (*ca*. 200 nm thick). The active area of the fabricated device was 3 × 3 mm^2^. For comparative purposes, we also fabricated two reference OPV cells using a vacuum-evaporated Ag anode (thickness: 100 nm) on the MoO_3_ HCL (ref. 1) and using a spin-coated AgNW anode (thickness: *ca*. 200 nm) on the MoO_3_ HCL (ref. 2). Apart from the differences in the Ag anodes described, the reference OPV cells were fabricated using exactly the same method as that used for the sample OPV cell with the H-dip-coated AgNW anode.

PV performance was measured using a source meter (2400, Keithley) and calibrated using a reference cell (BS-520, Bunkoh-keiki) under an illumination of 100 mW/cm^2^ produced by an AM 1.5G light source (96000 Solar Simulator, Newport). The reported values were averaged from several (at least 10) individual cells. The external quantum efficiency (EQE) spectra were obtained using a measurement system (Oriel^®^ IQE-200™ EQE/IQE, Newport).

## Results and Discussion

Figure 1a shows a meniscus of an AgNW suspension during the H-dip-coating process. With a Landau-Levich meniscus, the film thickness (*h*) can be described in terms of the capillary number (*C*_*a*_ = *μU/σ*) of the solution, by the associated drag-out problem; *h* = *k*· *C*_*a*_^2/3^·*R*_*d*_, for *C*_*a*_ ≪ 1, where *μ*, *σ*, *U*, *R*_*d*_, and *k* represent the viscosity, surface tension, coating speed, radius of the meniscus, and constant of proportionality, respectively^36^,^37^,^38^,^39^,^40^. We first investigated the film thickness of the H-dip-coated AgNWs (Fig. 1b). It is clear that for a given *h*_*0*_, the thickness of the AgNWs showed a continuous increase with increasing *U*. These results were in good agreement with the associated drag-out problem.

Figure 1c shows typical examples of homogeneous and transparent H-dip-coated AgNWs (thickness ~ 60 nm) formed on a 10 × 10 cm^2^ glass substrate. The figure also shows a SEM image of H-dip-coated AgNWs. Interestingly, nearly all the AgNWs are aligned unidirectionally along the direction of coating (*x*-direction) with few deviations, in contrast with randomly assembled AgNWs formed by spin-coating (Supplementary Fig. S1a). The surface morphology results obtained by AFM show that the values of the rms surface roughness for 40, 60, and 200 nm-thick H-dip-coated AgNWs were 12.8, 18.4, and 26.0 nm, respectively, which were almost identical at different positions on the investigated layers, while the rms surface roughness observed for the 40-nm-thick spin-coated AgNWs was ~22.1 nm. We then investigated the optical microscopic properties of the AgNWs under a polariser (the inset in the right panel of Fig. 1c): the observed texture is bright-state when the *x*-direction of the AgNWs is perpendicular to the transmission axis (P) of the polariser, but becomes dark-state when the *x*-direction is parallel to the P axis, indicating the clear dichroic properties of the AgNWs. These results confirm the two in-plane principal axes of the H-dip-coated AgNWs, where the *x*-direction is the light-absorbing axis and the *y*-direction is the polarising axis.

In contrast with previous techniques such as the spreading evaporation method^30^, the orientation of the H-dip-coated AgNWs is mainly homogeneous over the coated area. During the H-dip-coating process, the capillary fluid in the Landau-Levich meniscus near the substrate induces a shear stress^43^, particulary where the suspension passes under the coating barrier. The shear strain rate (γ) can be estimated by^43^ γ = *U*_fluid_/δ = ~50/s, where *U*_fluid_ is the capillary flow velocity near the bottom surface of the barrier, which is similar to *U* (~2 cm/s), and δ is the fluid depth at the edge (~*h*_0_). For *μ* = ~6.6 cp, the corresponding shear stress is τ = γ*μ* = ~0.33 Pa, which may be sufficient to achieve for the AgNW alignment. The Peclet number (*Pe*) for the AgNW suspension can also be estimated by^44^ *Pe* = *L*^3^γ*μ*/*kT* ~ 2.1 × 10^6^ ≫ 1, where *L* is the AgNW length (~30 μm) and *kT* is the thermal energy, implying that the motion of the AgNWs during H-dip-coating is dominated by hydrodynamic behaviour rather than by diffusion. Subsequently, the ordered AgNWs in the suspension gradually sink on to solid substrate, and finally a unidirectional alignment of AgNWs remains on the substrate after the solvent has evaporated. In addition, large deviations in a few AgNWs with respect to the coating direction can be attributed to insufficient rotation of the AgNWs adjacent to the substrate.

**Figure 2:**
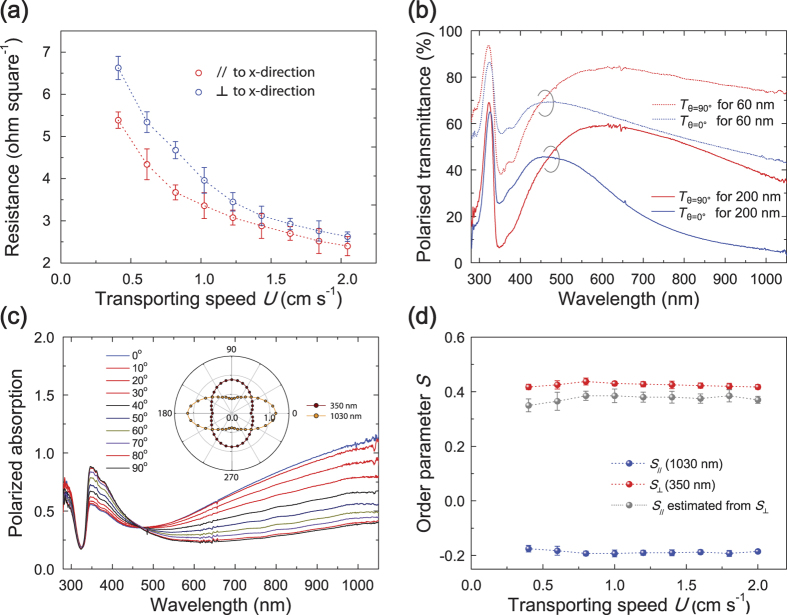
(**a**) Electric sheet resistance data measured parallel (//) and perpendicular (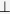) to the coating direction (*x*-direction) for the H-dip-coated AgNW films as a function of coating speed *U*. (**b**) Polarised UV-Vis transmission spectra of the H-dip-coated AgNW films (60 and 200 nm). The spectra were obtained at a polarisation angle *θ* of 0^o^ (*T*_θ__=__0°_, //, blue) and 90^o^ (*T*_θ__=__90°_, 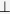, red), respectively. (**c**) Polarised UV-Vis absorption spectra of the H-dip-coated 200 nm-thick-AgNW film as a function of the polarisation angle *θ*. The inset shows the polar plot of the angular absorbance curves of the AgNW film for λs = 350 and 1030 nm. (**d**) Orientational order parameters *S*s of the H-dip-coated AgNW films, estimated from the absorptions at 350 nm (*S*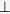) and 1030 nm (*S*_//_), as a function of coating speed *U*.

We then investigated the electrical anisotropy of the AgNWs by measuring the sheet resistances *R*_s_. Figure 2a shows that *R*_s_ for the AgNWs decreases with increasing *U*. For a given *U*, *R*_s_ measured along the *x*-direction is lower than that along the *y*-direction. While this *R*_s_ anisotropy is obvious for film thicknesses in the range <~170 nm (*U* = 1.5 cm/s), it is only slight for film thicknesses >~170 nm, which may be due to increasing inter-nanowire connectivity for thick AgNWs. In contrast, the optical anisotropy remains clear even for thick AgNWs. Figure 2b shows the polarised transmission spectra of the AgNWs, obtained at polarisation angles *θ* of 0^o^ and 90^o^, respectively. Here *θ* is defined as the angle between the polarisation direction of the incident light and the *x*-direction of the AgNWs. The polarised transmissions clearly show the prominent anisotropies of both the thin (60 nm) and thick (200 nm) AgNWs: in the long wavelength region (λ > 500 nm), the transmittance observed at *θ* = 0° (*T*_θ__=__0°_) is much lower than that observed at *θ* = 90° (*T*_θ__=__90°_). For short wavelengths (λ < 500 nm), *T*_θ__=__90°_ is higher than *T*_θ__=__0°_ for both AgNWs. To gain a better understanding of the optical anisotropy we observed the polarised absorption of the 200 nm-thick AgNW film. The AgNW film has a significant anisotropy of absorption (Fig. 2c), apart from near an isosbestic-like point at ~473 nm: in the short-wavelength region (e.g., λ = 350 nm), the polarised absorption for incident light polarised along the *y*-direction (*A*_θ__=__90°_) is stronger than it is for incident light polarised along the *x*-direction (*A*_θ__=__0°_), while *A*_θ__=__90°_ is weaker than *A*_θ__=__0°_ in the long-wavelength region, e.g., λ = 1030 nm. Here, the absorption peak at 350 nm is mainly attributable to the transverse SP resonances (SPRs) of the AgNWs, while the strong signals in the long-wavelength region over 1000 nm (peak at around 3100 nm, not shown) may be attributable to the longitudinal SPRs of the AgNWs^6^,^45^,^46^. Such optical anisotropy is characterised by the dichroic ratio *DR*, defined as^46^ *DR* = *A*_θ__=__0°_/*A*_θ__=__90°_. The measured dichroic ratios are *DR*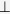 = 0.58 for the transverse (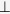) SPRs at λ=350 nm and *DR*_//_=3.18 at λ=1030 nm (for the longitudinal SPRs). From these *DR*s, we estimated the macroscopic orientational order parameter *S* of the AgNWs, defined as the statistical average (< >) of the second Legendre polynomial, i.e., *S* = <*P*_2_(cos *ϕ*)> = 1/2 (3 < cos^2^ *ϕ* >−1) for an angle *ϕ* between the long-wire axis of a single AgNW and the preferred direction^22^,^46^; *S* can be determined using the relationships: *S*_*//,*_ 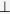 = (*DR*_*//,*_ 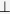 − 1)/(*DR*_*//,*_ 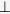 + 2) and *S*_*//*_ = −2 *S*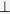, where *S*_*//*_ and *S*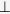 are the major order parameters with respect to the *x*- and *y(z)*-direction, respectively, for uniaxial symmetry^46^. Values of *S* estimated for AgNWs are shown in Fig. 2d, in which the degree of nanowire ordering shows obvious high values of *S*_*//*_ = ~0.38 for the observed range of *U*. Note that the *S*_*//*_ values (~0.38) estimated from *DR*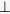 at λ = 350 nm can be confirmed by those (*S*_*//*_ = ~0.43) estimated from *DR*_//_ at λ = 1030 nm, resembling a nematic ordering (*S* = ~0.3–0.9) of archetypal liquid crystal (LCs) materials^46^. This *S*_*//*_ value of the H-dip-coated AgNWs is much higher than that (~0.0) of spin-coated AgNWs (Supplementary Fig. S1b), and is one of the highest values reported for oriented AgNWs^22^.

**Figure 3:**
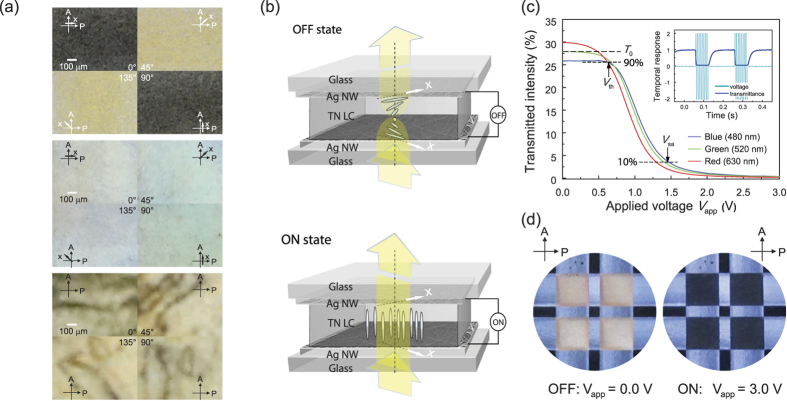
(**a**) Polarising microscopic textures of three nematic LC cells with a pair of transparent H-dip-coated AgNW films (60 nm) on glass substrates, whose coating directions are parallel (upper) and perpendicular (middle) to each other, respectively, and with a pair of transparent spin-coated AgNW films (60 nm) on glass substrates (lower) at four angles of rotation of the cells. The *x*-directions indicate the H-dip-coating direction of the AgNWs on the upper glass substrate, and the polarisation axes of the crossed polarisers are shown by the crossed arrows (**A,P**). (**b**) Schematic views of a twisted-nematic (TN) LC cell with a pair of transparent LC-alignment electrodes of AgNWs for two polarisation states of transmitted light in an OFF (upper) and an ON state (lower) with voltage applied. The polarisation of the incident light (yellow arrows) lies on the plane of the yellow arrows. (**c**) Transmittance of a TN-LC cell with transparent LC-aligning AgNW electrodes as a function of applied voltage (*V*_app_), operating in the normally white (NW) mode for blue, green, and red incident light. The inset shows the temporal responses of the TN-LC cell. (**d**) Photographs of operating 2 × 2 TN-LC cells with the LC-aligning AgNW electrodes in the NW mode for two applied bias voltages. The active area of each TN-LC cell is 3 × 3 mm^2^.

To address the need for large-scope applications in polarisation-selective opto-electronic devices, the H-dip-coating method can be used to apply the oriented AgNWs directly to a target substrate without any additional steps such as the transfer method^27^. As an example showing the use of H-dip-coated AgNWs, we investigated LC devices with oriented AgNWs as the transparent electrode in place of a conventional ITO electrode. Figure 3a shows the polarising microscopic textures of three nematic LC cells containing the AgNW electrodes. When the *x*-directions of the two H-dip-coated AgNW electrodes on glass substrates are in parallel, the nematic LC molecules are homogeneously (or planar) aligned on the oriented AgNWs in the *x*-direction due to the free energies of the anisotropic surface (Supplementary Fig. S2), even in the absence of any other alignment layer or treatment (upper panel in Fig. 3a). Interestingly, when the *x*-directions of the two H-dip-coated AgNW electrodes are perpendicular, the nematic LC molecules between the oriented AgNW electrodes form a 90° twist of the LC director, i.e., a TN structure, resulting in a 90° rotation of the polarisation of the light after it passes through the cell, as in waveguide mode^47^ (middle panel in Fig. 3a). In contrast, when the two spin-coated AgNWs on glass substrates were used as electrodes, the nematic LC molecules were inhomogeneously aligned on the randomly assembled AgNWs (lower panel in Fig. 3a). Hence, when H-dip-coated AgNW electrodes are used in LC cells, various LC modes are possible, depending on the combination of the oriented AgNW electrodes, which act not only as a transparent conductive electrode but also as an LC-alignment layer (Fig. 3b).

Next, we measured the electro-optic characteristics of the TN-LC cell with the AgNWs as a function of the voltage applied (*V*_*app*_) under the NW mode^47^ (Fig. 3c). It can be seen that for the voltage-off state (or *V*_*app*_ < threshold voltage, *V*_*th*_, ~ 0.70 V), the transmittance is high (the absolute transmittance, *T*_*0*_ ~ 25–30%), representing the bright state (Fig. 3c). In contrast, for *V*_*app*_ > saturation voltage (*V*_*sa*_) ~ 1.45 V, the effective retardation value decreases due to the field-induced re-orientation of LCs between the AgNW electrodes, and the amount of transmitted light decreases. Thus, the contrast ratio (*CR*) of the intensity of bright to dark increases as *V*_*app*_ increases, giving maximum *CR* values of *ca*. 78.3, 91.7, and 175.9 for R, G, and B light, respectively. Moreover, the rising (field on) and falling (field off) times of the TN-LC cell were found to be approximately 3.4 and 33.9 ms, respectively. Such a low *V*_*th*_ and high *CR*, and such fast switching times, make the AgNW TN-LC cell suitable for a variety of video-rate display applications. In addition, in the NB mode the operation of the TN-LC cell is inversely related to *V*_*app*_ (Supplementary Fig. S3). These results show the considerable performance advantages of the LC-alignment electrodes of AgNWs in the LC devices.

**Figure 4:**
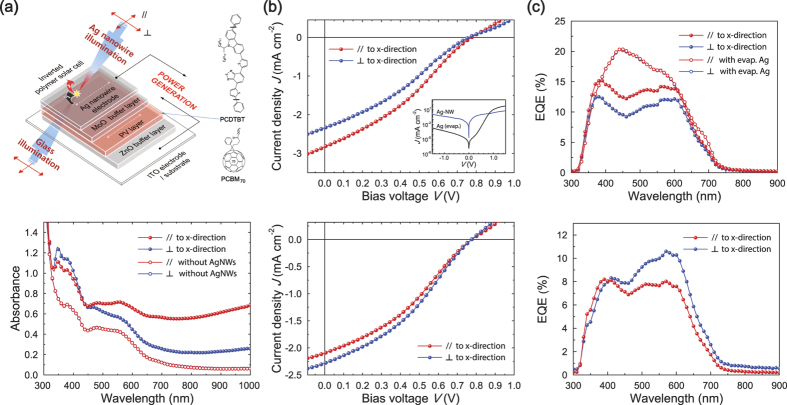
(**a**) Upper: Schematic structure of a (semi-)transparent inverted OPV cell containing a transparent H-dip-coated AgNW anode (sample AgNW-OPV cell). The red arrows indicate the polarisations of the incident lights for illumination from glass-side and AgNW anode-side, and the white dotted arrow shows the *x*-direction of the AgNWs. Lower: Polarised UV-Vis absorption spectra of a semi-transparent inverted PCDTBT:PCBM_70_ OPV cell with (closed circles) and without (open circles) an oriented AgNW anode for incident light polarised parallel (//) and perpendicular (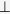) to the *x*-direction. (**b**) Polarisation-dependent *J–V* characteristics of the sample PCDTBT:PCBM_70_ OPV cells studied under polarised illuminations from glass-side (upper panel) and AgNW anode-side (lower panel). The inset shows semilogarithmic plots of the performance of the OPV cells in the dark. (**c**) EQE spectra of the OPV cells with the oriented AgNW anodes (or with the evaporated Ag anode) under polarised illuminations from glass-side (upper panel) and AgNW anode-side (lower panel).

Another use of the H-dip-coated AgNWs is a transparent polarising electrode in an opto-electonic device, e.g., an OPV cell. Here, we also investigated (semi-)transparent OPV cells having oriented AgNWs, whose structure and energy diagram are shown in Fig. 4a and Supplementary Fig. S4a, respectively. In view of the transparency of the OPV cells, either the glass substrate side (glass illumination) or the AgNW anode side (AgNW illumination) can be subject to light irradiation. The oriented AgNW anode may also serve as a polarisation-selective optical window for incident light. The PV layer in the OPV cells may be either isotropic or anisotropic (e.g., rubbed^48^), but isotropic BHJ PV layers of low-band-gap PCDTBT:PCBM_70_ were used in our case to investigate the effect of the oriented AgNW anode on device performance. Note that the PCDTBT:PCBM_70_ layer is too soft to endure conventional rubbing for generating anisotropy, in contrast to a conventional OPV layer^48^.

Figure 4a also shows the polarised absorption of the PCDTBT:PCBM_70_ OPV cell with the H-dip-coated AgNW anode (sample AgNW-OPV cell). The sample AgNW-OPV cell exhibited two strong broad absorption bands with peaks at 398 and 576 nm caused by the PCDTBT, extending to an absorption onset at 720 nm, together with an absorption at *ca.* 450 nm caused by the PCBM_70_. In contrast to the isotropic absorptions of the PCDTBT:PCBM_70_ PV layer (Fig. 4a) and the spin-coated AgNW electrode (Supplementary Fig. S1b), the sample AgNW-OPV cell with the H-dip-coated AgNW anode shows clear absorption anisotropy due to the oriented AgNW anode, which selectively absorbs and transmits (or reflects, Supplementary Fig. S4b) incident lights polarised parallel and perpendicular to the *x*-direction, respectively.

We then investigated the current density-voltage (*J–V*) characteristics of the OPV cells. In the dark, the reference PV cell with an evaporated Ag anode clearly revealed good diodic behaviour with high rectification ratios of 10^4^ at 1.5 V, while the sample AgNW-OPV cell exhibited similar but slightly different current flows (Fig. 4b), which may have been due to differences in the internal resistance and variations in the interfacial potential barrier between the PV layers and the anodes. The PV characteristics of the sample and reference OPV cells were investigated using unpolarised and polarised illumination, and their performances are summarised in Supplementary Tables S1 and S2. When tested under unpolarised glass illumination, the sample AgNW-OPV cell with the 85 nm-thick PCDTBT:PCBM_70_ PV layer and the H-dip-coated AgNW anode achieved a power conversion efficiency (PCE) of 2.22%, with a short-circuit current density (*J*_SC_) of 9.35 mA/cm^2^, an open-circuit voltage (*V*_OC_) of 0.82 V, and a fill factor (*FF*) of 29.08%, while when tested under unpolarised Ag-illumination, the cell showed a PCE of 1.99%, with a *J*_SC_ of 8.13 mA/cm^2^, a *V*_OC_ of 0.82 V, and an *FF* of 29.85% (Supplementary Table S1 and Fig. S5). For comparison, under unpolarised glass illumination, ref. 1 with the vacuum-evaporated Ag anode and the 85 (65) nm-thick PV layer achieved a PCE of 3.81 (4.51)%, which is comparable to values (*ca*. 4.9%) reported previously^42^, while ref. 2 with the spin-coated AgNW anodes achieved a PCE of 1.72% under unpolarised glass illumination and a PCE of 1.49% under unpolarised Ag illumination.

The polarisation-dependent *J-V* characteristics of the OPV cells were then investigated (Fig. 4b and Supplementary Table S2). Under glass illumination, the sample AgNW-OPV cell gave a clear anisotropic PV performance: for polarised incident light along the *x*-direction, the sample cell with the 85 nm-thick PV layer exhibited a PCE of about 2.49%, which is much higher than results (*ca*. 1.94%) for light polarised along the *y*-direction. This may have been caused by the polarised reflection of the oriented AgNW anode (Supplementary Fig. S4b). Thus it is clear that the sample AgNW-OPV cell showed a considerable degree of anisotropy in terms of the PV effects (PCE_//_/PCE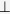 = 1.28). Interestingly, under AgNW illumination, the sample AgNW-OPV cell showed a reverse anisotropy of PV performance (PCE_//_/PCE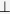 = 0.90), which may be attributed to the polarised absorption of the oriented AgNW anodes (Fig. 4a). In contrast, ref. 2 with the spin-coated AgNW anode showed clear isotropic PV effects under both illuminations, i.e., PCE_//_/PCE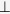 ≈ 1.0 (Supplementary Table S2). It is also noteworthy that the anisotropic *J*_SC_ behaviours of the sample cells are consistent with their EQE spectra (Fig. 4c): the polarised reflection spectra of the oriented AgNW anode (Supplementary Fig. S4b) are responsible for the EQE spectral shapes under glass illumination (Fig. 4c), while the absorption spectra of the OPV layer (Fig. 4a) together with the polarised transmission spectra of the oriented AgNW anode (Fig. 2b) are responsible for the EQE spectral shapes under Ag illumination. The foregoing results clearly show that the sample AgNW-OPV cell with the H-dip-coated AgNW anode acted as a transparent polarising OPV cell, providing clear evidence of anisotropic PV activity even for isotropic PV layers. We infer that further improvements to the PV anisotropy might be expected by incorporating anisotropic PV layers^48^ in place of an isotropic PV layer in the sample AgNW-OPV cell, and a more detailed investigation of this will be presented elsewhere.

**Figure 5:**
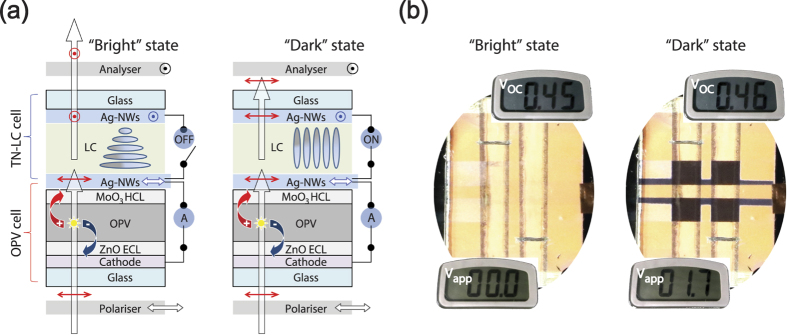
(**a**) Schematic illustration of the bright (left) and dark (right) states of the transmission-type Solar-LC cells, consisting of linear dichroic polarisers, TN-LC cells, and transparent polarising PCDTBT:PCBM_70_ OPV cells, in which the oriented AgNW electrodes serve for both the TN-LC and OPV cells, in the NW mode. The white arrows indicate the propagation of the incident lights and the red arrows represent the polarisation directions of the propagating lights. The black arrows and circled dots indicate the passing axes of the polarisers. (**b**) Photographs of power-generating PCDTBT:PCBM_70_ Solar-LC cells (2 × 2 pixels), displaying bright (left-hand panel) and dark (right-hand panel) states in the NW mode for two applied voltages (*V*_app_) of 0.0 V and 1.7 V, respectively. The active area of each TN-LC cell is 3 × 3 mm^2^.

Finally, as an example to show the combined effects of our findings, using the H-dip-coated AgNWs we fabricated and tested a power-generating transmission-type TN-LC device by imbedding a polarising OPV cell, i.e., a Solar-LCD with a schematic structure, as shown in Fig. 5a. Figure 5b shows an operating Solar-LCD, displaying bright and dark states in the NW mode, and simultaneously generating electricity from the backlight illumination. This example also clearly shows that the H-dip-coated AgNW electrode can serve not only as an LC-alignment electrode but also as a polarising, hole-collecting electrode in a Solar-LCD.

Considering all these results, it is clear that oriented AgNWs produced by H-dip-coating can act as a multi-functional anisotropic optical and electrical component in various polarising opto-electronic devices.

## Conclusions

In summary, we investigated homogeneous and uniaxial ordering of H-dip-coated AgNWs for large-area polarising printed/organic electronics. Self-assembled and well oriented AgNWs obtained via the simple H-dip-coating process were fabricated with a significantly high order parameter of ~0.38–0.43 due to capillary flow in the meniscus of suspension. We showed that oriented AgNWs can be used in LC devices as a transparent LC-aligning electrode, exhibiting good LC device performance. Moreover, oriented AgNWs can also be used in inverted OPV cells as a transparent polarising anode, exhibiting high PV anisotropy. These results for well oriented AgNWs fabricated by H-dip-coating provide an encouraging basis for the design and fabrication of large-area, flexible, low-cost, high-performance, and polarisation-dependent applications in advanced printed/organic optoelectronics.

## Additional Information

**How to cite this article**: Park, B. *et al.* Aligned silver nanowire-based transparent electrodes for engineering polarisation-selective optoelectronics. *Sci. Rep.* **6**, 19485; doi: 10.1038/srep19485 (2016).

## Supplementary Material

Supplementary Information
