# Supplementary material for: Aligned silver nanowire-based transparent electrodes for engineering polarisation-selective optoelectronics
Source: Sci Rep. 2016 Jan 18;6:19485. doi: 10.1038/srep19485 (PMC4725987; doi:10.1038/srep19485)
Supplement: Supplementary Information [file srep19485-s1.doc]

SUPPLEMENTARY INFORMATION

Aligned silver nanowire-based transparent electrodes for engineering polarisation-selective optoelectronics

Byoungchoo Park^*^, In-Gon Bae, and Yoon Ho Huh

Department of Electrical and Biological Physics, Kwangwoon University,

Wolgye-Dong, Nowon-gu, Seoul 139-701, Republic of Korea

*e-mail: [bcpark@kw.ac.kr](mailto:bcpark@kw.ac.kr)

(Supplementary Information)


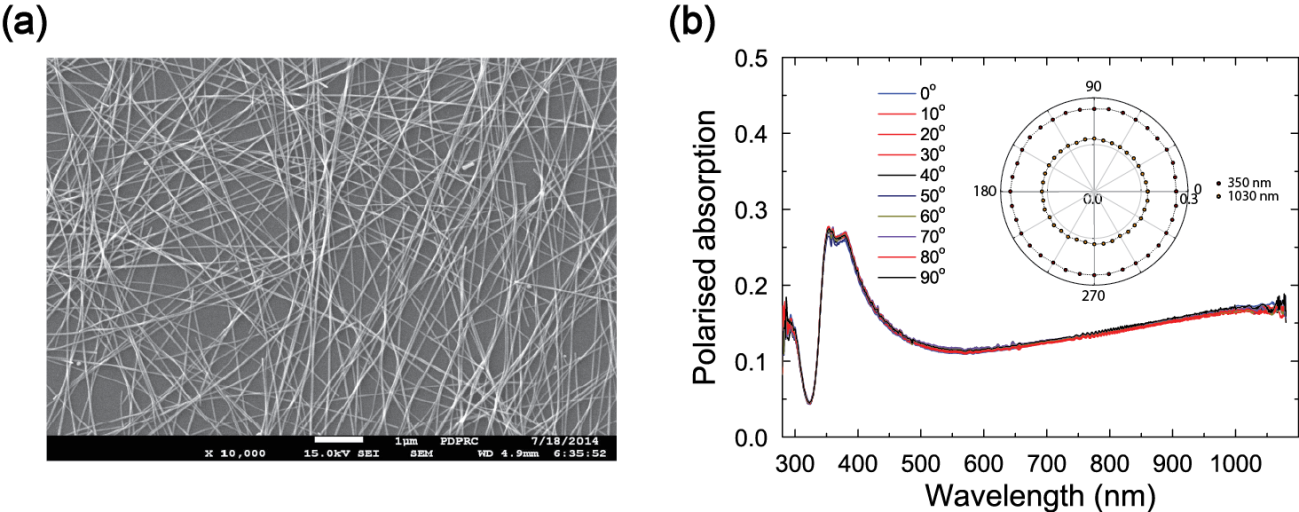


Figure S1. Microscopic image of a spin-coated AgNW film and its optical absorption. (a) SEM image of a spin-coated AgNW film on a glass substrate. (b) Polarised UV-Vis absorption spectra of the spin-coated AgNW film as a function of polarisation angle *θ*.


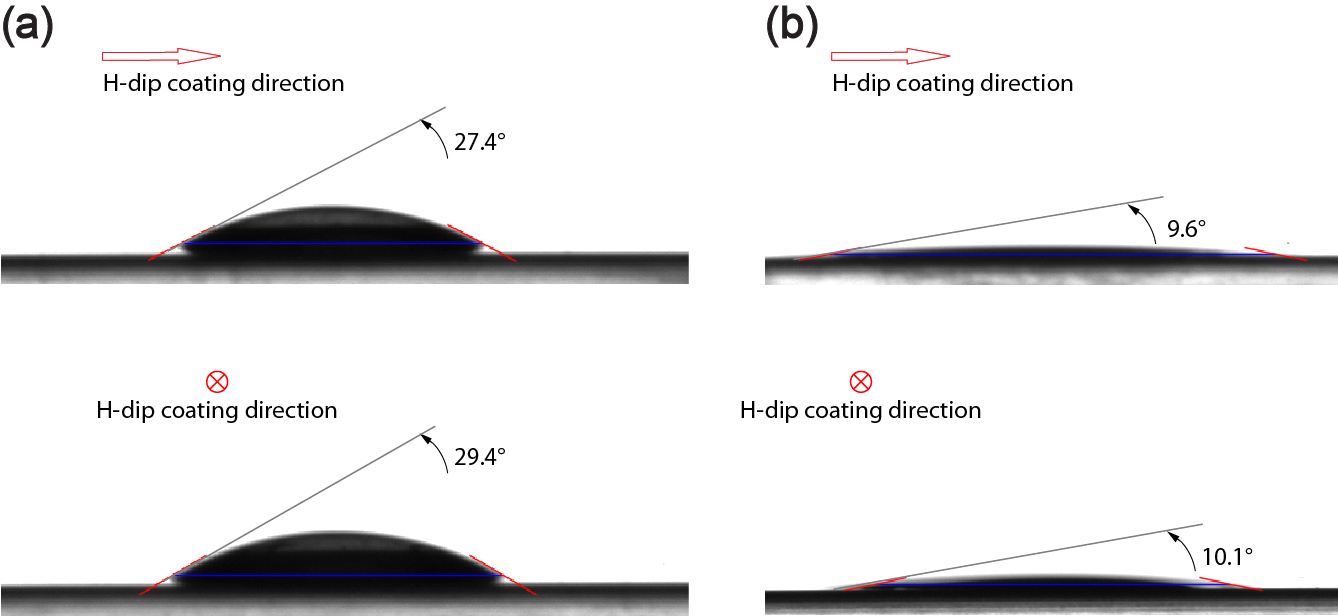


Figure S2. Contact angles of H-dip-coated AgNWs. Static contact angles of deionised water (a) and toluene (b) droplets on the H-dip-coated AgNW films (~40 nm) along the directions parallel (upper) and perpendicular (lower) to the H-dip-coating directions.

Figure S2 shows the observed static contact angles of deionised water and toluene droplets on the H-dip-coated AgNW films (~40 nm) along the directions parallel and perpendicular to the H-dip-coating directions. As shown in Fig. S2, the H-dip-coated AgNW film shows obvious anisotropic wettabilities. The direction parallel to the H-dip-coating directions (contact angles: 27.4° for DI water and 9.6° for toluene) is much more wettable than that perpendicular to the H-dip-coating directions (contact angles: 29.4° for DI water and 10.1° for toluene), clearly indicating the anisotropic surface free energy of the AgNW films: in our case, the surface free energy (~65.8 mJ/m^2^) in the H-dip-coating directions is higher than that (~64.8 mJ/m^2^) perpendicular to the H-dip-coating directions, which similarly occurs for the conventional rubbed polyimide alignment layers^S1^. However, in contrast to the strong anisotropic chemical interactions between the LC molecules and the conventional rubbed polymer surface, we attribute the anisotropic physical affinity due to the surface topography of the oriented AgNWs to the alignment of the LC molecules.


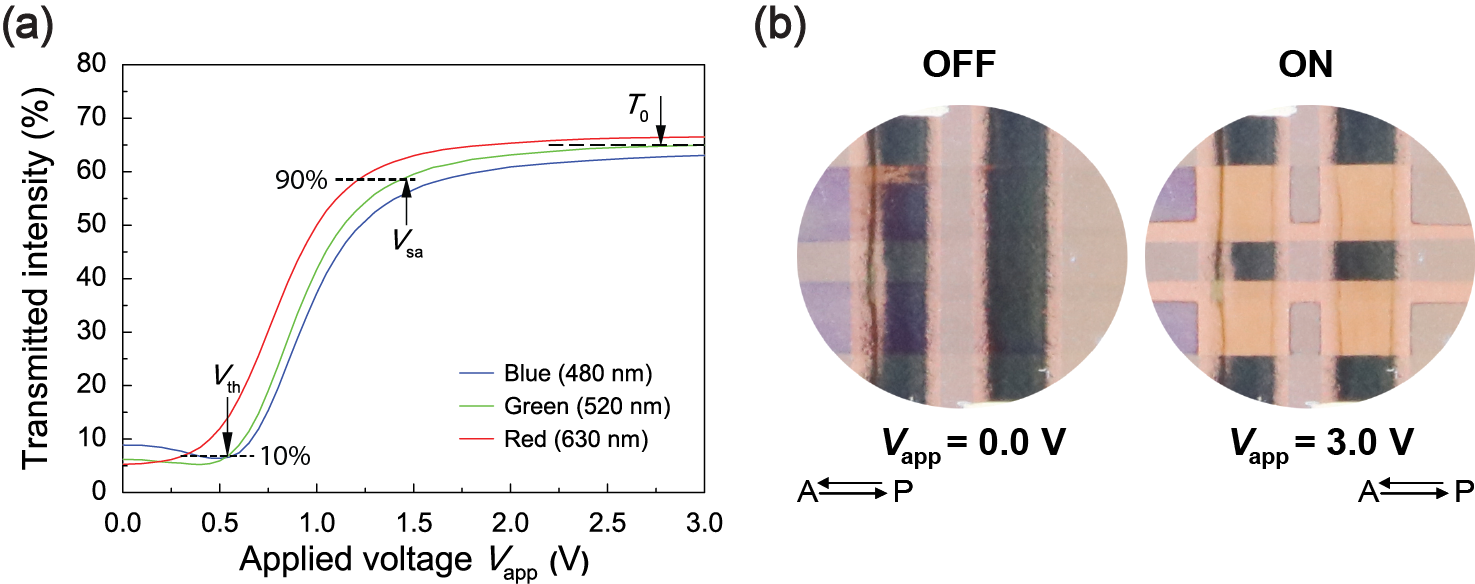


Figure S3. Electro-optic responses of an AgNW TN-LC cell. (a) Transmittance of a TN-LC cell with a pair of transparent LC-aligning AgNW electrodes, as a function of applied voltage *V_app_*, operating in the normally black (NB) mode for blue (480 nm), green (520 nm), and red (630 nm) incident light. Maximum *CR* values are *ca*. 8.9, 11.4, and 12.1 for B, G, and R light, respectively. (b) Photographs of operating 2 × 2 TN-LC cells with the LC-aligning AgNW electrodes in the NB mode at two bias voltages applied (*V_app_*). The active area of each TN-LC cell is 3 × 3 mm^2^.


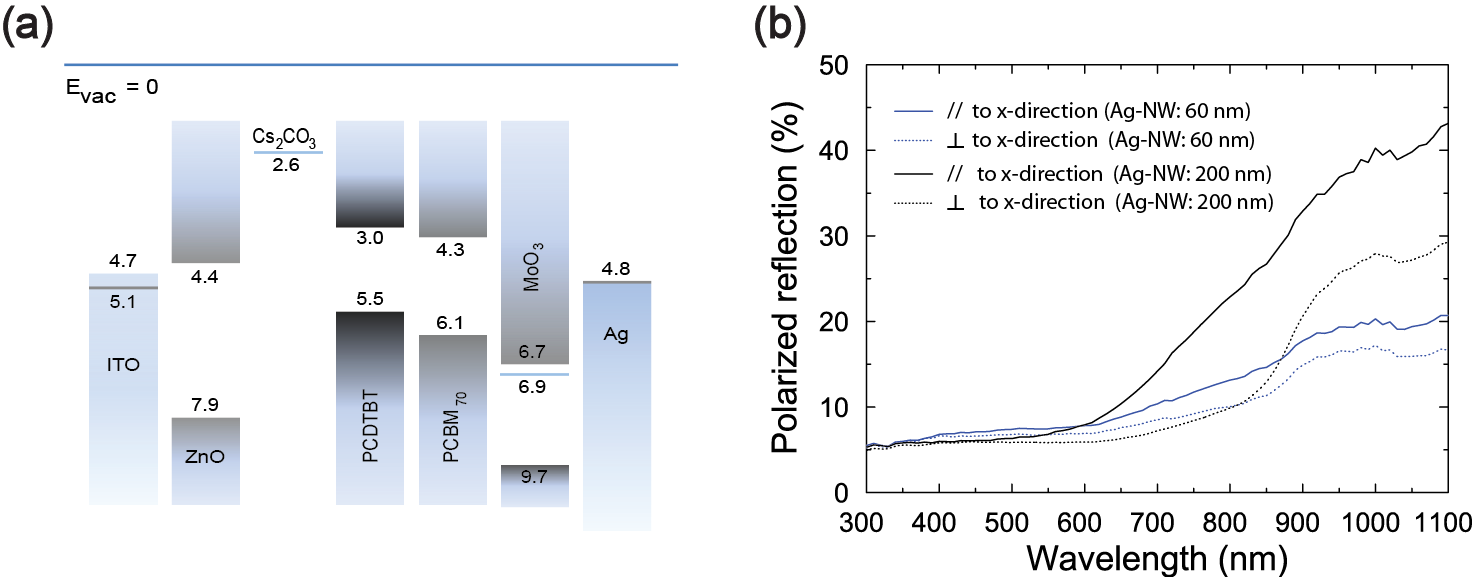


Figure S4. Energy diagram of the OPV cells studied and polarised optical reflections of the AgNWs used. (a) The relevant energy level diagrams of the PCDTBT:PCBM_70_ OPV cells studied. (b) Polarised reflectance spectra of the H-dip-coated AgNW electrodes (60 and 200 nm).


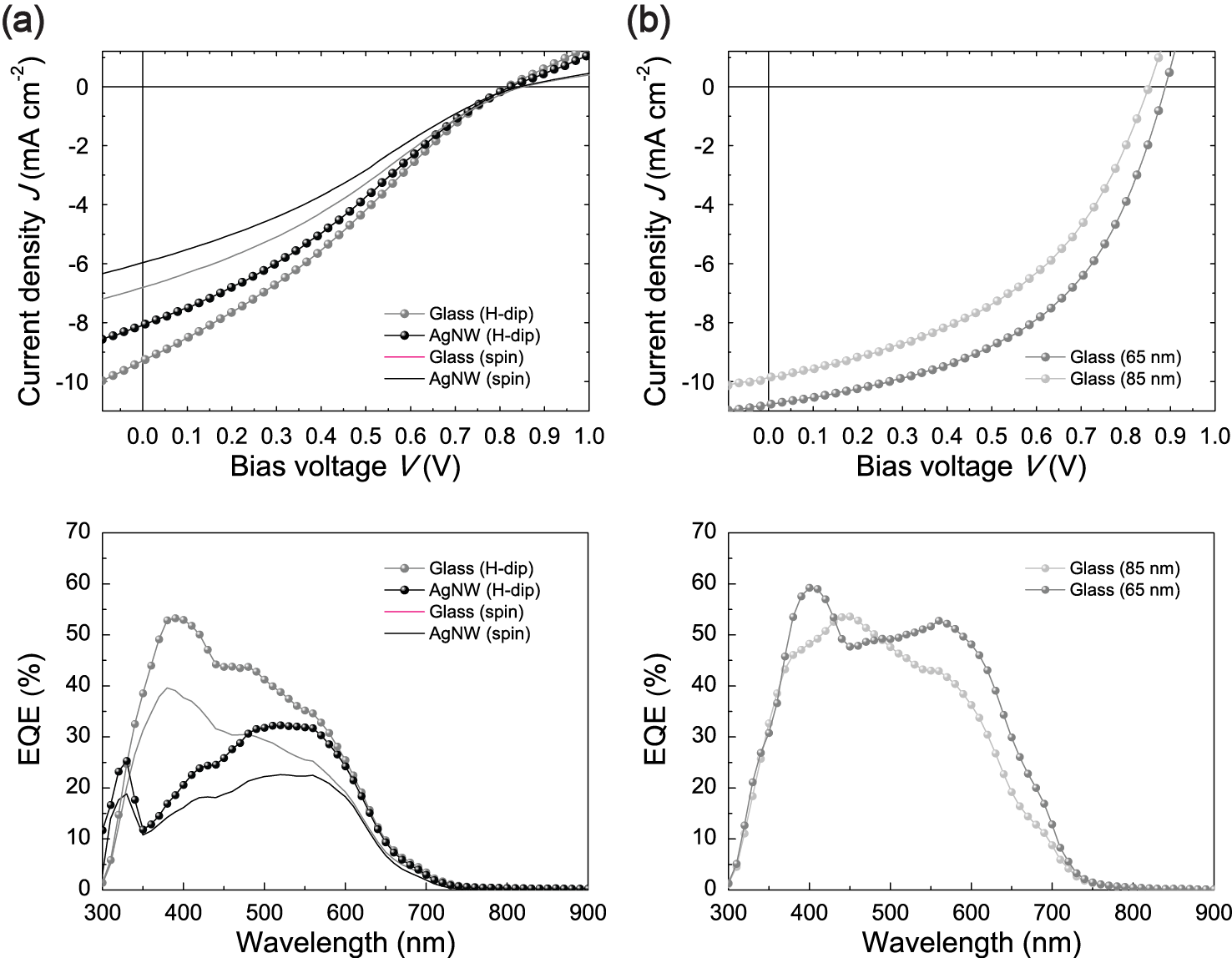


Figure S5. Photovoltaic characteristics of OPV cells studied. (a) Representative *J-V* (upper) and EQE (lower) characteristics of the OPV cells with the H-dip-coated (sample) or the spin-coated (reference 2) AgNW anodes and the 85-nm-thick PCDTBT:PCBM_70_ PV layers under illumination from glass-side and AgNW anode-side. (b) Representative *J-V* (upper) and EQE (lower) characteristics of the OPV cell (reference 1) with the evaporated Ag anode and the 85- or 65-nm-thick PCDTBT:PCBM_70_ PV layers under illuminations only from glass-side. Note that, under Ag illumination, reference 1 gave no PV performance because of the highly reflective and opaque Ag electrode used.

**Table S1. Summary of device performance of inverted PCDTBT:PCBM_70_ OPV cells under unpolarised illumination from glass-side and anode-side.**

| Anodes | PV layer  thickness | Irradiation direction | *J*_sc_ (mA/cm^2^) | *V*_oc_ (V) | *FF* (%) | PCE (%) |
| --- | --- | --- | --- | --- | --- | --- |
| Sample:  H-dip-coated AgNWs | 65 nm | Glass | 7.70±0.16 | 0.84±0.01 | 28.52±0.57 | 1.85±0.07 |
|  |  | AgNWs | 6.76±0.20 | 0.84±0.00 | 29.99±0.82 | 1.70±0.07 |
|  | 75 nm | Glass | 9.28±0.29 | 0.83±0.01 | 27.66±0.30 | 2.14±0.06 |
|  |  | AgNWs | 7.83±0.07 | 0.84±0.00 | 28.74±0.32 | 1.88±0.03 |
|  | 85 nm | Glass | 9.35±0.21 | 0.82±0.01 | 29.08±0.64 | 2.22±0.09 |
|  |  | AgNWs | 8.13±0.20 | 0.82±0.01 | 29.85±0.88 | 1.99±0.09 |
|  | 95 nm | Glass | 8.42±0.51 | 0.81±0.01 | 26.61±1.11 | 1.82±0.08 |
|  |  | AgNWs | 6.96±0.53 | 0.83±0.02 | 26.55±2.17 | 1.52±0.06 |
| Reference 1: Evaporated Ag | 65 nm | Glass | 10.70±0.13 | 0.85±0.01 | 49.45±0.80 | 4.51±0.12 |
|  | 75 nm | Glass | 10.01±0.08 | 0.86±0.00 | 48.70±0.74 | 4.20±0.09 |
|  | 85 nm | Glass | 9.88±0.25 | 0.85±0.00 | 45.36±0.63 | 3.81±0.13 |
|  | 95 nm | Glass | 9.17±0.30 | 0.85±0.01 | 44.70±1.07 | 3.47±0.09 |
| Reference 2:  Spin-coated AgNWs | 85 nm | Glass | 6.81±1.16 | 0.85±0.02 | 29.83±1.41 | 1.72±0.24 |
|  |  | AgNWs | 5.93±1.10 | 0.85±0.02 | 29.70±1.50 | 1.49±0.24 |

Average values were obtained from at least ten devices.

**Table S2. Summary of device performance for inverted PCDTBT:PCBM_70_ OPV cells under illumination polarised parallel (//) and perpendicular (┴) to the *x*-direction from glass-side and anode-side.**

| Anodes | PV layer thickness | Irradiation Direction | State of  polarisation | *J*_sc_ (mA/cm^2^) | *V*_oc_ (V) | *FF* (%) | PCE (%) | PCE Ratio |
| --- | --- | --- | --- | --- | --- | --- | --- | --- |
| Sample:  H-dip-coated AgNWs | 65 nm | Glass | // | 2.25±0.23 | 0.78±0.02 | 32.01±1.04 | 1.97±0.19 | 1.20±0.05 |
|  |  |  | ┴ | 2.07±0.21 | 0.78±0.03 | 29.06±1.72 | 1.65±0.19 |  |
|  |  | Anode | // | 1.74±0.17 | 0.77±0.02 | 33.55±0.89 | 1.57±0.14 | 0.90±0.02 |
|  |  |  | ┴ | 1.90±0.19 | 0.77±0.02 | 34.09±0.77 | 1.74±0.15 |  |
|  | 75 nm | Glass | // | 2.53±0.13 | 0.80±0.01 | 31.76±0.44 | 2.27±0.13 | 1.22±0.02 |
|  |  |  | ┴ | 2.25±0.10 | 0.80±0.01 | 29.61±1.09 | 1.86±0.10 |  |
|  |  | Anode | // | 1.92±0.14 | 0.79±0.01 | 32.36±1.81 | 1.71±0.15 | 0.87±0.01 |
|  |  |  | ┴ | 2.15±0.18 | 0.79±0.01 | 32.92±1.87 | 1.96±0.18 |  |
|  | 85 nm | Glass | // | 2.85±0.15 | 0.78±0.01 | 31.93±0.83 | 2.49±0.17 | 1.28±0.08 |
|  |  |  | ┴ | 2.36±0.10 | 0.78±0.01 | 30.02±1.94 | 1.94±0.15 |  |
|  |  | Anode | // | 2.10±0.06 | 0.77±0.01 | 34.09±1.23 | 1.94±0.11 | 0.90±0.04 |
|  |  |  | ┴ | 2.29±0.06 | 0.77±0.01 | 34.61±1.34 | 2.15±0.11 |  |
|  | 95 nm | Glass | // | 2.69±0.20 | 0.78±0.01 | 29.54±1.96 | 2.18±0.11 | 1.24±0.07 |
|  |  |  | ┴ | 2.32±0.23 | 0.78±0.01 | 27.73±2.37 | 1.76±0.14 |  |
|  |  | Anode | // | 2.03±0.09 | 0.77±0.01 | 32.17±1.36 | 1.77±0.10 | 0.91±0.05 |
|  |  |  | ┴ | 2.22±0.10 | 0.77±0.01 | 32.43±1.20 | 1.95±0.11 |  |
| Reference 1: Evaporated Ag | 65 nm | Glass | // | 2.85±0.07 | 0.83±0.00 | 54.35±1.41 | 4.48±0.07 | 1.00±0.01 |
|  |  |  | ┴ | 2.87±0.07 | 0.82±0.00 | 54.20±1.30 | 4.49±0.10 |  |
|  | 75 nm | Glass | // | 2.76±0.05 | 0.82±0.01 | 53.23±0.77 | 4.23±0.04 | 1.00±0.01 |
|  |  |  | ┴ | 2.77±0.04 | 0.82±0.01 | 53.58±1.11 | 4.25±0.04 |  |
|  | 85 nm | Glass | // | 2.65±0.15 | 0.80±0.01 | 48.29±1.81 | 3.59±0.08 | 1.00±0.01 |
|  |  |  | ┴ | 2.65±0.14 | 0.80±0.01 | 48.48±1.62 | 3.61±0.09 |  |
|  | 95 nm | Glass | // | 2.49±0.09 | 0.80±0.01 | 47.76±1.35 | 3.35±0.10 | 1.00±0.01 |
|  |  |  | ┴ | 2.49±0.10 | 0.80±0.01 | 47.88±1.42 | 3.35±0.10 |  |
| Reference 2:  Spin-coated AgNWs | 85 nm | Glass | // | 1.78±0.31 | 0.82±0.02 | 33.03±1.41 | 1.67±0.22 | 1.01±0.02 |
|  |  |  | ┴ | 1.77±0.32 | 0.82±0.02 | 33.05±1.37 | 1.66±0.23 |  |
|  |  | Anode | // | 1.65±0.40 | 0.80±0.02 | 30.36±1.49 | 1.39±0.28 | 0.99±0.03 |
|  |  |  | ┴ | 1.66±0.38 | 0.80±0.01 | 30.39±1.28 | 1.41±0.27 |  |

Average values were obtained from at least ten devices.

**Supplementary references**

1. Zheng, W. [Surface wetting characteristics of rubbed polyimide thin films] Polymer thin films [Hashim, A. A. (ed.)] [161-184] (InTech, Rijeka, 2010).
